# The Need for Specialism

**Published:** 1914-02-14

**Authors:** 


### THE NEED FOR SPECIALISM.

By M. O.

Professor Harvey Cushing's Address in Surgery at the International Congress of Medicine probably contained more that is suggestive and stimulating for the general body of medical practitioners than any other single contribution to the Congress. He emphasised the inability of the practitioner to be chemist, neurologist, bacteriologist, ophthalmologist, radiographer, and surgeon too, and instanced a case of cerebral gumma as probably requiring the services of all these specialists. His case is that the individual practitioner is not and cannot be adequately equipped to meet satisfactorily the needs of his patients. If such be the case now, how much more so will it be true in the not distant future! If Professor Cushing's argument can be sustained, then it is full time for the profession boldly to face the facts and devise proper means for meeting the altered conditions.

The day is not so far distant when there were physicians and surgeons who exercised their energies on all parts of the body. As one well-known general surgeon of the old school was wont to say: "The laryngologist has come down from above and the gynæcologist has come up from below, leaving us only a small area round the umbilicus. And there are the navel surgeons, gentlemen." But to-day there are still finer distinctions. The services of clinical pathologists are in growing demand and, indeed, are essential to the modern treatment of innumerable diseases. Discharges, excreta and body fluids, the blood and cerebrospinal fluid, contain in them the secrets of many ailments; attempts at diagnosis and treatment without their examination are largely empirical. Medicine is becoming more and more an exact science, and the laboratory is an essential part of medical equipment. The days of the bottle and the spoon, as Professor Cushing points out, are rapidly passing away.

"Team" Work.

There are even specialists among laboratory workers. Histologists, bacteriologists, and biochemists have enough to do in their own fields without trespassing too much on the province of their co-workers, and there can be no doubt that the system of "team" work by a series of investigators working at different aspects of the same problem as carried out at the Rockefeller Institute, and as suggested by many to the Departmental Committee on Tuberculosis, yields and promises to yield results far more quickly and more surely than the unorganised system at present too commonly in vogue. The radiologist is another worker whose services are being increasingly required for diagnosis and, if Professor Rist, of Paris, is to be believed, the *x*-rays are as necessary in the diagnosis of chest affections as the stethoscope itself.

As Professor Cushing has shown, diagnosis may call for the co-operation of several clinical experts in special fields, specialists in the eye, ear, brain, or operative surgery. Similarly in treatment the aid of all those specialists may be required. The sanatorium scheme has called into being numbers of men calling themselves tuberculosis experts, and soon the services of a considerable body of men skilled in the treatment of syphilis and venereal diseases generally will be required. No doubt cancer will follow suit.

It is clear that in the near future there will be a great extension of the clinical laboratory system. Most of the big counties and the bigger towns of 50,000 population and over already possess them, and the area of their activities is ever extending. All kinds of clinical materials are being reported on in them, usually free of cost to the practitioner and to the advantage of the public. The early development and extension of this system throughout the country is therefore to be desired. The doors of these laboratories should be thrown widely open, and practitioners should be encouraged not only to use them to the fullest extent, but to visit them and find in them a centre of inspiration and knowledge. At present practitioners do under great difficulties endeavour to keep themselves abreast of progress by visiting the hospitals at intervals, but nowhere could they so easily keep in touch with the science of medicine as in the public clinical laboratories in their own districts.

But there will also be a still further differentiation of function in clinical work, and here we have a problem calling for the most earnest consideration of all members of the profession. Though there are in this country many eminent men engaged in the various specialties, it cannot be said that their number is adequate for the need. It is notoriously true that men of very limited experience have been appointed as tuberculosis experts, and there is certainly an insufficiency of men competent to undertake the modern diagnosis and treatment of syphilis. There is, therefore, an urgent need for the training of men to undertake special services. As a corollary to this it is equally clear that the demand for differentiation in function should be recognised and arrangements made to meet it not only in the curriculum of students, but also in practice. Recruits for special services cannot continue to be drawn from among recently qualified men, and if the need for specialism is recognised and admitted it is not too late for the general body of practitioners to allot themselves or be allotted in their several districts to the various branches of medicine to which they might devote their special attention and study, and for the practice of which—among insured persons, for instance—their services might be availed of by their colleagues. Reform has come from without, and possibly against our will, as in the case of the Insurance Act. In the present instance there is no reason why it should not come from within and be demanded by ourselves.

